# Supplementary material for: Immunomodulatory and Antioxidant Properties of Wheat Gluten Protein Hydrolysates in Human Peripheral Blood Mononuclear Cells
Source: Nutrients. 2020 Jun 4;12(6):1673. doi: 10.3390/nu12061673 (PMC7352691; doi:10.3390/nu12061673)
Supplement: Supplementary file 1 [file nutrients-12-01673-s001.pdf]

Supplemental Table S1. Sequences of primers used for real-time PCR analysis

Target gene	Primer sequences	PCR product (bp)	T annealing (°C)
Cytokine			
IL-10<br>NM_000572.3	Fwd 5′-CAC GGC CTT GCT CTT GTT TT-3′<br>Rev 5′-GTG ATG CCC CAA GCT GAG A-3′	138	54
Antioxidant enzymes			
CAT<br>NM_001752.4	Fwd 5′-TAA GAC TGA CCA GGG CAT C-3′<br>Rev 5′-CAA ACC TTG GTG AGA TCG AA-3′	201	56
GPX<br>NM_000581.4	Fwd 5′-AGT CGG TGT ATG CCT TCT CGG-3′<br>Rev 5′-GTT CTT GGC GTT CTC CTG ATG-3′	227	60
GR<br>NM_000637.5<br>NM_001195102.2	Fwd 5′-GCC CTG GGT TCT AAG ACA TCA-3′<br>Rev 5′-CTC AGG TCC TTG GTA TTC GGG-3′	275	56
SOD<br>NM_000454.5	Fwd 5′-AGG TGT GGG GAA GCA TTA-3′<br>Rev 5'-TGG TCT CCT GAG AGT GAG ATC-3′	241	56
Nitric Oxide-producer			
INOS<br>NM_000625.4	Fwd 5′- TGC AGA CAC GTG CGT TAC TCC -3′<br>Rev 5'- GGT AGC CAG CAT AGC GGA TG -3′	130	60
Housekeeping gene			
B-ACTIN<br>NM_001101.5	Fwd 5′-AGA GCT ACG AGC TGC CTGAC-3′<br>Rev 5′-AGC ACT GTG TTG GCG TACAG-3′	184	54 - 60

Names of the genes with accession numbers, forward (Fwd) and reverse (Rev) sequences of the primers, numbers of base pairs (bp) of PCR products and annealing temperatures. CAT, catalase; GPX, glutathione peroxidase; GR, glutathione reductase; IL-10, interleukin-10; INOS, inducible nitric oxide synthases; SOD, superoxide dismutase; B-ACTIN, beta-actin.

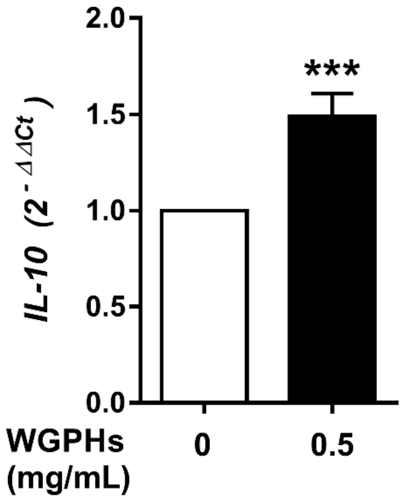

Supplemental Fig. S1. Relative gene expression of IL-10 in PHA-stimulated PBMCs after overnight treatment with 0.5 mg/mL WGPHs. The data represent the mean of the value calculated with respect to PHA-stimulated, untreated cells with the 2-ΔΔCt method, and the standard error of the mean of each group (n= 9). *** p≤0.001 with respect to the control group (WGPHs 0 mg/mL).
